# Follow-Up 18F-FDG PET/CT versus Contrast-Enhanced CT after Ablation of Liver Metastases of Colorectal Carcinoma—A Cost-Effectiveness Analysis

**DOI:** 10.3390/cancers12092432

**Published:** 2020-08-27

**Authors:** Moritz L. Schnitzer, Matthias F. Froelich, Felix G. Gassert, Thomas Huber, Eva Gresser, Vincent Schwarze, Dominik Nörenberg, Andrei Todica, Johannes Rübenthaler

**Affiliations:** 1 Department of Radiology, University Hospital, LMU Munich, Marchioninistr. 15, 81377 Munich, Germany; Moritz.Schnitzer14@gmail.com (M.L.S.); eva.gresser@med.uni-muenchen.de (E.G.); Vincent.Schwarze@med.uni-muenchen.de (V.S.); 2 Department of Clinical Radiology and Nuclear Medicine, University Medical Center Mannheim, Theodor-Kutzer-Ufer 1-3, 68167 Mannheim, Germany; Matthias.Froelich@medma.uni-heidelberg.de (M.F.F.); Thomas.Huber@medma.uni-heidelberg.de (T.H.); Dominik.Noerenberg@medma.uni-heidelberg.de (D.N.); 3 Department of Diagnostic and Interventional Radiology, Klinikum rechts der Isar, Technical University of Munich, Ismaninger Str. 22, 81675 Munich, Germany; felix.gassert@tum.de; 4 Department of Nuclear Medicine, University Hospital, LMU Munich, Marchioninistr. 15, 81377 Munich, Germany; Andrei.Todica@med.uni-muenchen.de

**Keywords:** 18F-FDG PET/CT, oncologic imaging, cost-effectiveness, liver metastases, nuclear imaging

## Abstract

Purpose: After a percutaneous ablation of colorectal liver metastases (CRLM), follow-up investigations to evaluate potential tumor recurrence are necessary. The aim of this study was to analyze whether a combined 18F-Fluordesoxyglucose positron emission tomography-computed tomography (18F-FDG PET/CT) scan is cost-effective compared to a contrast-enhanced computed tomography (CE-CT) scan for detecting local tumor progression. Materials and Methods: A decision model based on Markov simulations that estimated lifetime costs and quality-adjusted life years (QALYs) was developed. Model input parameters were obtained from the recent literature. Deterministic sensitivity analysis of diagnostic parameters based on a Monte-Carlo simulation with 30,000 iterations was performed. The willingness-to-pay (WTP) was set to $100,000/QALY. Results: In the base-case scenario, CE-CT resulted in total costs of $28,625.08 and an efficacy of 0.755 QALYs, whereas 18F-FDG PET/CT resulted in total costs of $29,239.97 with an efficacy of 0.767. Therefore, the corresponding incremental cost-effectiveness ratio (ICER) of 18F-FDG PET/CT was $50,338.96 per QALY indicating cost-effectiveness based on the WTP threshold set above. The results were stable in deterministic and probabilistic sensitivity analyses. Conclusion: Based on our model, 18F-FDG PET/CT can be considered as a cost-effective imaging alternative for follow-up investigations after percutaneous ablation of colorectal liver metastases.

## 1. Introduction

Colorectal cancer (CRC) is among the most prevalent tumor malignancies worldwide. Every tenth diagnosed cancer is attributable to a colorectal tumor. With nearly 900,000 deaths per year, regarding mortality it is within the top four cancer entities worldwide [1]. Approximately 50% of patients with CRC will be diagnosed with metastases either at the time of diagnosis or as part of recurrent disease where the liver is the most common site for metastases [2]. The liver is the most common site of hematogenous metastatic spread in patients with CRC with 50–60% of patients with CRC developing liver metastases [3]. In daily clinical practice, surgical resection has emerged as the treatment of choice for solitary liver metastases given that these patients achieve 5-year survival rates above 50% [2]. However, only 25% of affected patients are suitable to undergo a surgical procedure due to size, number and localization of metastases [2]. Limiting factors for resection of liver metastases are critical locations within the liver such as close location to the big vessels, impaired liver function and multi-comorbidity. For the remaining majority of patients who are not suitable for surgery, several alternative, minimally invasive treatment options such as percutaneous ablation, for example radiofrequency ablation (RFA) or microwave ablation (MWA), are already applied in clinical routine and have been shown to be efficient alternatives in treatment of these patients [4,5]. Overall, untreated liver metastases are lethal for almost all patients by 5 years as liver metastases come with a median survival of only 8 months [6]. Particularly in liver lesions smaller than 3 cm, RFA combined with chemotherapy is a considerable alternative to conventional surgical treatment for both palliative and curative intentions [7,8]. Although the successful ablation rates have improved in the last years, percutaneous ablation treatment results in locoregional recurrence in up to 40% of patients, and consequently close radiological surveillance is necessary for early detection of local tumor recurrence [9,10]. Depending on the regarding reports, 5-year survival rates show a variability of 14% to 55% [11]. Accurate staging is of the utmost importance for guiding the most suitable therapeutic options and follow-up investigation is crucial for patients’ survival to preclude possible local tumor progressions (LTP) [12]. At this juncture, several diagnostic techniques such as CE-CT (contrast-enhanced computed tomography), ultrasound (US), magnetic resonance imaging (MRI) and 18F-FDG-PET/CT (18F-Fluordesoxyglucose positron emission tomography-computed tomography) are used in the clinical routine for detection of local recurrence after ablation [13]. Preceding studies showed that 18F-FDG PET/CT has a higher sensitivity compared to CE-CT, which is along with MRI the imaging modality of choice for evaluation of treatment efficacy [12]. Two meta-analyses showed that 18F-FDG-PET/CT has the highest diagnostic accuracy for the diagnosis of hepatic metastases from tumors with colorectal, gastric or esophageal primary cancer origin [14,15]. However, due to its higher costs, the cost-effectiveness of 18F-FDG PET/CT for daily use in detection of colorectal liver metastases is still questioned. The existing literature on the clinical use of 18F-FDG PET/CT in CRC is rather limited although it is suggested that the use of 18F-FDG PET/CT can lead to a change in primary intended treatment strategy in up to 30% of patients. Some guidelines also emphasize the effectiveness of 18F-FDG PET/CT in patients with consecutively elevated levels of carcinoembryonic antigen level (CEA) or in patients with potential false-negative results on conventional imaging [16], as elevated levels of CEA cannot provide accurate localization to a potential site of recurrence [17]. Notwithstanding, CE-CT is still being considered as the primary imaging modality of choice for staging and re-staging due to its comparatively low cost, ubiquitous availability, fast image acquisition time and high anatomical resolution. The main disadvantage of conventional CE-CT for assessing potential recurrence after percutaneous ablation is the inability to sufficiently differentiate between recurrent neoplastic tissue and postinterventional nonmalignant changes, such as scars, inflammation or potential necrosis, and the potential underestimation of clinically relevant tumor burden by missing small tumor clusters in postinterventional altered anatomy [18]. 18F-FDG-PET/CT is considered to have a higher diagnostic accuracy compared to conventional imaging methods in the early assessment of potential local recurrence and metastases as well as being superior in evaluation of tumor viability after local intervention [19,20,21,22].

In order to assess and potentially overcome high treatment costs, the aim of this study was to systematically analyze the cost-effectiveness of 18F-FDG PET/CT and CE-CT and for CRLM patients after percutaneous ablation.

## 2. Results

### 2.1. Estimated Outcomes and Corresponding Costs

**Figure 1:**
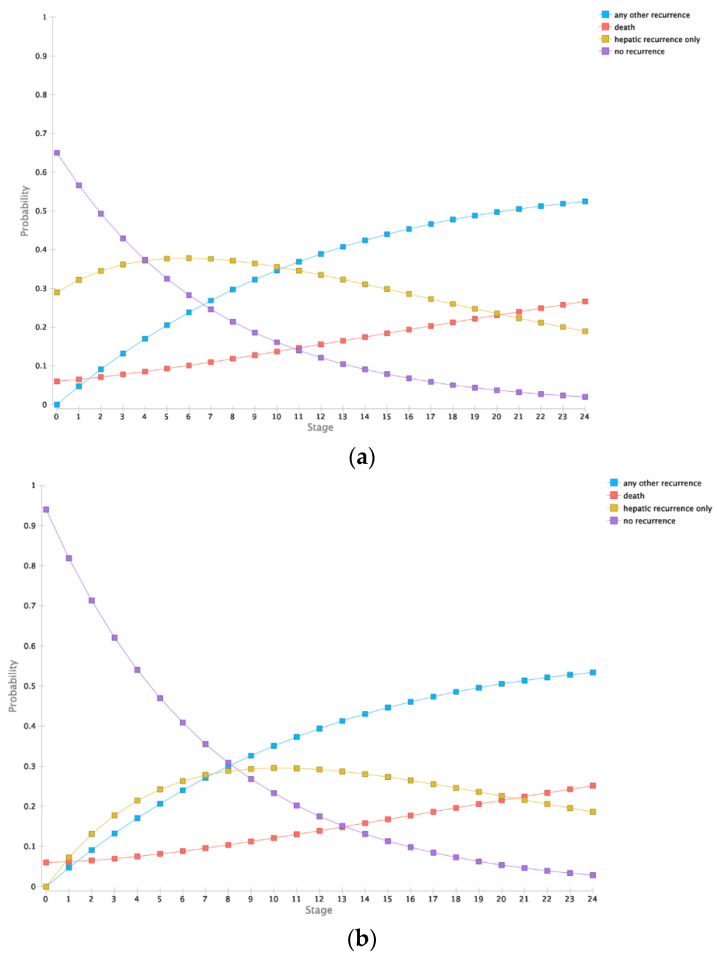

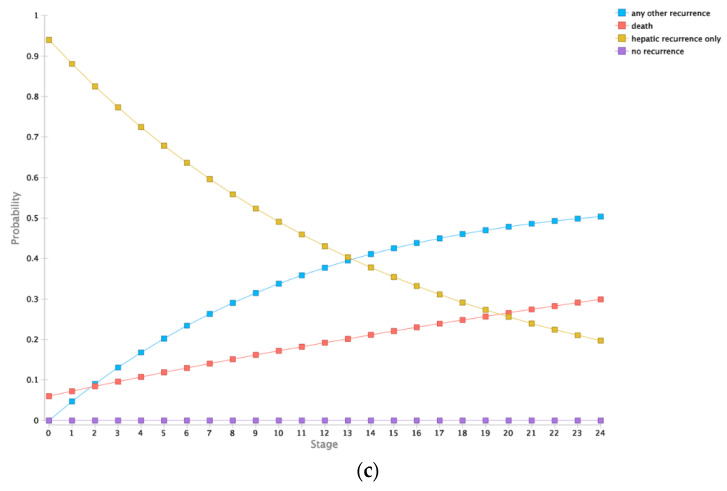
Markov simulation for two years. (**a**). Outcomes for patients receiving a timely reablation (true positive). (**b**). Outcomes of patients without initially needed reablation (true negative and false positive). (**c**). Outcomes of patients with delayed reablation (false negative).

Outcomes were estimated in a Markov model as described below. Therefore, patients without evidence of an active hepatic tumor (true negative and false positive group) and patients with a successfully detected and, therefore, timely ablated lesion (true positive group) were modelled comparably. After two years, true positive patients had expected cumulative costs of $35,395.51 and a cumulative quality of life of 0.741 QALYs (quality-adjusted life years). In comparison, the group with false negative findings resulted in cumulative costs of $41,927.60 and a cumulative quality of life of 0.611 QALYs. Furthermore, patients without initially indicated reablation showed a cumulative quality of life of 0.786 and cumulative costs of $23,979.07 and $34,208.07 for true negative (TN) and false positive (FP) respectively. Modelled overall survival in the FN group was 70% in contrast to 74.8% in TN group. The detailed per-month modelled states are summarized in Figure 1.

### 2.2. Cost-Effectiveness Analysis

Based on the results of the Markov model, a baseline cost-effectiveness analysis resulted for CE-CT in total costs of $28,625.08 and an efficacy of 0.755 QALYs, whereas 18F-FDG PET/CT resulted in total costs of $29,239.97 with an efficacy of 0.767. Therefore, the corresponding ICER (incremental cost-effectiveness ratio) of 18F-FDG PET/CT resulted in $50,338.96 per QALY.

### 2.3. Deterministic Sensitivity Analysis

**Figure 2:**
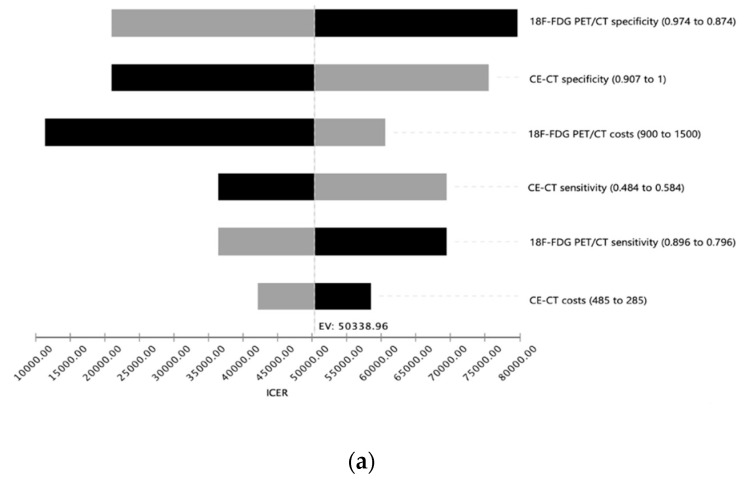

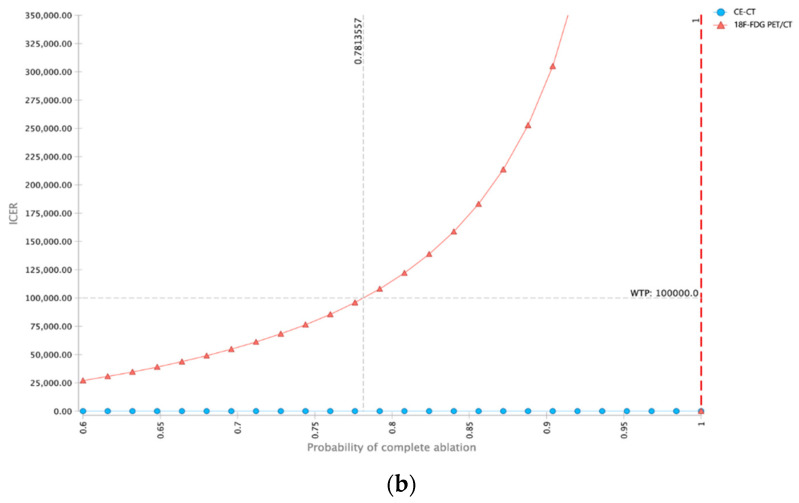
Deterministic sensitivity analysis. (**a**). Tornado diagram showing the impact of input parameters on incremental cost-effectiveness ratio (ICER) starting from expected value (EV) in base case scenario. For every parameter range investigated, the ICER of 18F-FDG PET/CT remained below the willingness-to-pay threshold of $100,000 per QALY (quality of life year) indicating cost-effectiveness of 18F-FDG PET/CT in this setting. (**b**). Analysis investigating the impact of probability of complete ablation on cost-effectiveness. Especially in patients with high risk of incomplete ablation, 18F-FDG PET-CT is the cost-effective strategy in comparison to CE-CT. ICER, incremental cost-effectiveness ratio; WTP, willingness-to-pay.

To investigate the stability of the model, a deterministic sensitivity analysis including costs, sensitivities and specificities of both diagnostic modalities was performed. Within the ranges applied, the ICER for 18F-FDG PET/CT remained below the WTP (willingness-to-pay) threshold of $100,000 per QALY indicating cost-effectiveness of 18F-FDG PET/CT (Figure 2a).

As the pre-test probability of incomplete ablation is a key measure in this patient collective, an additional deterministic sensitivity analysis focused on this parameter was performed. As would be expected, the ICER of 18F-FDG PET/CT as a diagnostic modality with increased diagnostic accuracy increased with a higher probability of complete ablation. The probability of 78.14% for complete ablation was determined as the threshold above which the ICER of 18F-FDG PET/CT was higher than the WTP threshold of $100,000 per QALY. Therefore, 18F-FDG PET/CT may be particularly cost-effective in patients with a higher risk of incomplete ablation.

### 2.4. Probabilistic Sensitivity Analysis

**Figure 3:**
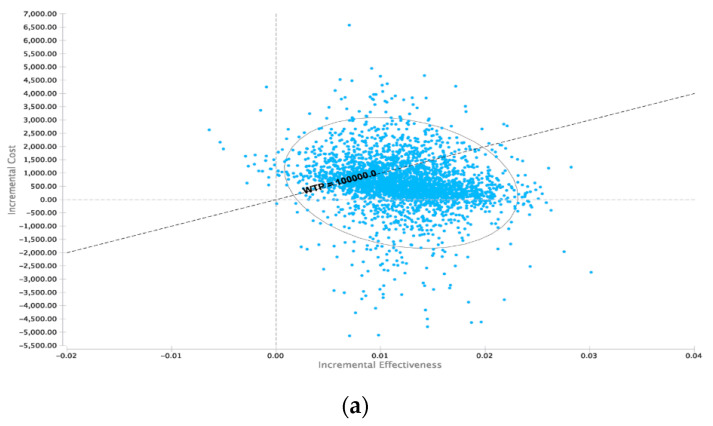

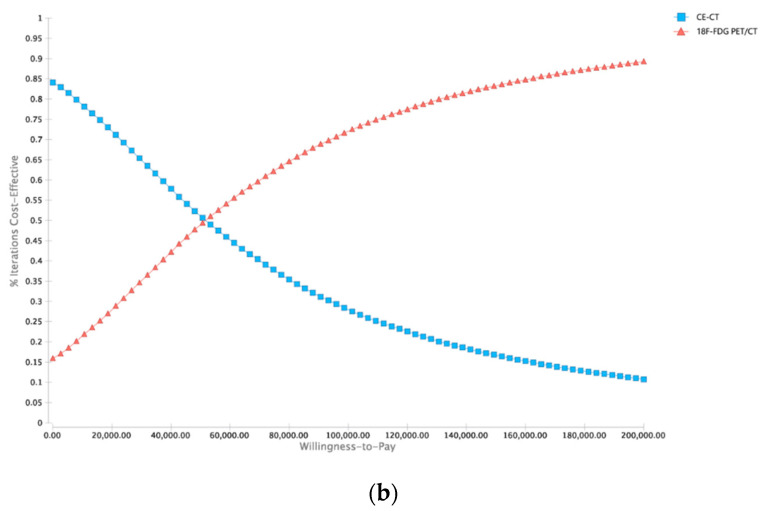
Probabilistic sensitivity analysis utilizing Monte-Carlo simulations with 30,000 iterations. (**a**). Incremental cost-effectiveness scatterplot (18F-FDG PET-CT vs. CE-CT). (**b**). Cost-effectiveness acceptability curve dependent on willingness-to-pay (WTP). 18F-FDG PET-CT is cost-effective in the majority of iterations above a WTP-threshold of $45,000.

To further investigate the robustness of the model, a probabilistic sensitivity analysis based on the distributions described in Table 1 was performed. At the WTP of $100,000 per QALY, 18F-FDG PET/CT was cost-effective in 71.96 % of iterations (Figure 3a). An increased WTP was associated with a higher proportion of iterations being cost-effective for 18F-FDG PET/CT (Figure 3b).

## 3. Discussion

In the post-ablative course, close surveillance and early re-intervention are necessary in order to achieve optimal ablation success and monitoring of therapy success is considered to be a main topic in interventional liver therapy. Many studies have reported on the diagnostic accuracy of CE-CT and 18F-FDG PET/CT. However, it is not entirely clear how 18F-FDG PET/CT in comparison to CE-CT could be a cost-effective strategy in solely colorectal cancer patients and how it could be incorporated into the routine post-ablation follow-up imaging algorithm. The limitations of conventional CE-CT as the primary imaging modality of choice in the detection of local recurrence after ablation in CRC patients with liver metastases include the reliance on solely morphological features leading to potential failure in the correct differentiation between postinterventional viable and non-viable tumor tissue potentially resulting in lower QALYs and higher long-term costs. These limitations can only be relatively compensated by the use of contrast media as therapy-induced hyperperfusion in the periphery of the ablation zone cannot be differentiated from viable tumor tissue in all cases. Recent studies already show that 18F-FDG PET/CT shows a higher diagnostic accuracy for the evaluation of hepatic metastases than conventional CE-CT imaging [32]. Some studies even demonstrate that 18F-FDG PET/CT can provide the same diagnostic accuracy for the assessment of hepatic metastases as MRI [21].

Our study demonstrates that 18F-FDG PET/CT may be a cost-effective strategy for follow-up investigations after ablation of colorectal liver metastases compared to CE-CT alone.

Although 18F-FDG PET/CT was cost effective in the base-case analysis, some limitations of this strategy need to be acknowledged. The results were strongly dependent on the risk of incomplete ablation as 18F-FDG PET/CT is a valuable possibility for the detection of incomplete ablated tumor margins which cannot be detected in CE-CT scans [33]. In a scenario where the operator skills improve and complete ablation of lesions is the general case, 18F-FDG PET/CT could lose cost-effectiveness. However, as long as incomplete ablation rates are still high, 18F-FDG PET/CT should definitively be considered for follow-up investigations based on these modelling results. Furthermore, inflammatory changes of hepatic tissue after ablation can distort the regular outcome of 18F-FDG PET/CT into a false-positive assessment [34]. Nonetheless, in scenarios with relatively big lesions or lesions with suspicion of incomplete ablation, 18F-FDG PET/CT should be considered as a cost-effective strategy.

**Figure 4:**
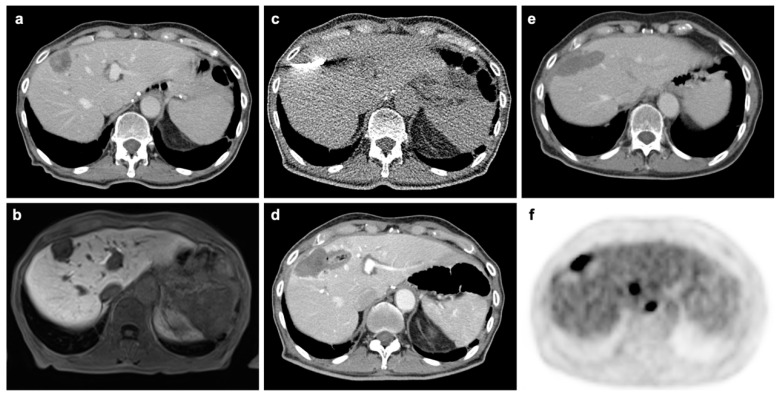
Patient from our institution undergoing microwave ablation (MWA) for oligometastatic liver disease. (**a**). Pre-interventional CT in portal-venous phase shows contrast-enhancing metastasis in segment VIII. (**b**). Pre-interventional MRI in hepatocyte-specific phase. (**c**). Interventional imaging during MWA. (**d**). CT in portal-venous phase acquired immediately after intervention without evidence of complication or residual tumor. (**e**). Four-month follow-up F-18 FDG PET/CT without morphological evidence of recurrence in CT component. (**f**). PET-component acquired at same scan 4-month after MWA with tracer accumulation at the margins of the previous ablation in line with recurrence. Additional metastases without morphological evidence in the CT component can also be depicted.

Our study is based on previous literature proving the advantages of 18F-FDG PET/CT in diagnostic performance in a meta-analysis including 10 studies with 304 patients [12]. By now, several other studies have published reports on the diagnostic performances of CE-CT and 18F-FDG PET/CT of this exact matter. As a matter of fact, sensitivity and specificity reported by other studies deviate from our utilized values. Thereby, using other values that differ substantially from our values, 18F-FDG PET/CT may lose cost-effectiveness. However, as the analysis we utilized is a larger meta-analysis, we decided to limit the used input parameters to one main source. As 18F-FDG PET/CT is able to detect metabolic alterations even before a tumor is even anatomically visible, it enables the physician to detect a remaining malignancy much earlier than with CE-CT [12]. Figure 4 shows the case of an example patient proving the advantage of 18F-FDG PET/CT as a recurrence can be seen that cannot be seen in CE-CT.

For achieving the most convenient outcome, a split-dose 18F-FDG PET/CT should be used immediately after ablation to guarantee a complete ablation [33,35,36], or within 24–48 h [4,37]. Future follow-up investigations should be performed in an interval of 2–4 months in order to provide timely discovery of tumor recurrence [38].

At the time of the investigation, there is already an analysis of 18F-FDG-PET, MRI and CE-CT after ablation on hepatic tumors including hepatic cell carcinoma (HCC), neuroendocrine tumors (NET), colon carcinoma and several other entities from 2013 that reports that 18F-FDG PET/CT is the cost-effective strategy [39]. Though these results are in accordance with our analysis, as there are variable types of tumors in this study, an exclusive analysis of colorectal metastasis is still necessary. Furthermore, there are several studies that surveyed cost-effectiveness of follow-up investigation in different tumor entities. First of all, a 2010 study proved cost-effectiveness of 18F-FDG PET/CT in the follow-up of non-small cell lung tumors after a radical radiotherapy combined with and without chemotherapy [40]. Moreover, a study in 2010 investigated the cost-effectiveness of 18F-FDG PET/CT in screening for distant metastases of head and neck tumors showing the superiority of 18F-FDG PET/CT examination [41]. Nonetheless, even 18F-FDG PET/CT has its limitations. For instance, a study in 2013 showed that 18F-FDG PET/CT is not a cost-effective approach for surveillance and follow-up after cervical cancer treatment as it provides only a minimal increase in effectiveness with a significant increase of costs [42], proving that 18F-FDG PET/CT is not a broadly applicable solution. Our analysis is in accordance with current literature regarding 2-year overall survival rates around 70% [43]. Median survival is reported to be between 33.3 and 59 months [38,43,44]. Although our Markov model is only designed for a 2-year simulation, it seems due to its similarity to the published data to be a valid result.

Our model is due to its nature very dependent on the input parameter we utilized in our analysis. Naturally, it never reflects the clinical reality, as several parameters are likely to deviate as the case arises. Especially in cases with a false negative outcome when patients’ suffering is not perceivable, the model is difficult as a pursuing treatment is not intended. In addition to that, the results are very dependent on the complete ablation success. With a complete ablation rate near 100%, 18F-FDG PET/CT would become redundant and not cost-effective. On the other hand, a decline of success rates of ablation would lead to increasing recurrence rates, which could have been detected early by a timely 18F-FDG PET/CT examination. This would justify an additional scan and entail cost-effectiveness. However, as published literature reports a deviation of ablation success between 86.7% and 94%, and those rates also depend on the attending physician, a case-by-case decision about 18F-FDG PET/CT may be necessary [38,45]. Therefore, an additional sensitivity analysis was performed constituting the sensitivity, specificity and costs of 18F-FDG PET/CT and CE-CT in detail. We consciously excluded MRI from our study because the published evidence does not yet offer a sufficient data foundation for a cost-effectiveness. As far as the related current literature reports, MRI is comparable to 18F-FDG PET/CT regarding its sensitivity and specificity [12].

Some healthcare professionals are worried that basing clinical decision-making on ICERs will reduce the types of treatments available to patients. It is important to emphasize that cost-effectiveness analyses are used to inform healthcare providers and key opinion leaders in the health care system but they must not do this automatically and consecutively lead to an individual diagnostic or therapeutic decision without cautiously considering all individual circumstances of each separate case of the relevant healthcare system. Similarly, commonly used WTP thresholds of $50,000 or $100,000 per QALY should not be considered as absolute borders and it is essential to point out that the optical medical threshold is a matter of sociopolitical willingness of the health care system rather than one of scientific or medical debate.

In conclusion, our study demonstrates that 18F-FDG PET/CT can be cost-effective as a follow-up diagnostic tool after ablation of CRLM especially in cases with a high risk of incomplete ablation due to relatively big lesions or lesions which are more difficult for the operator to excise. Under these circumstances, 18F-FDG PET/CT is as a matter of fact an economical approach for follow-up investigations in a daily clinical routine.

## 4. Materials and Methods

### 4.1. Model Structure

**Figure 5:**
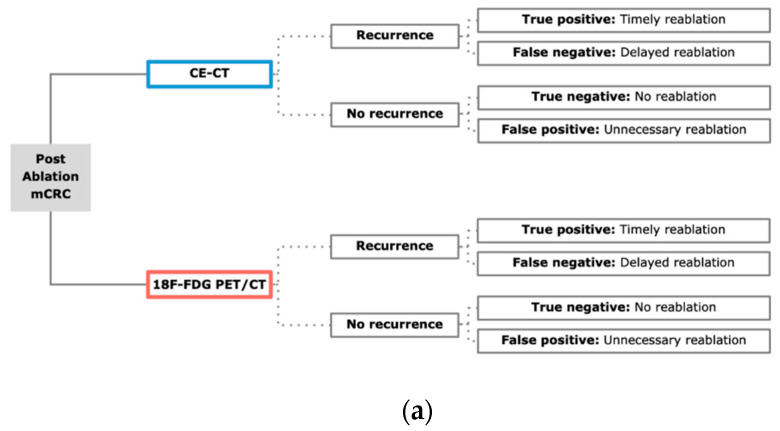

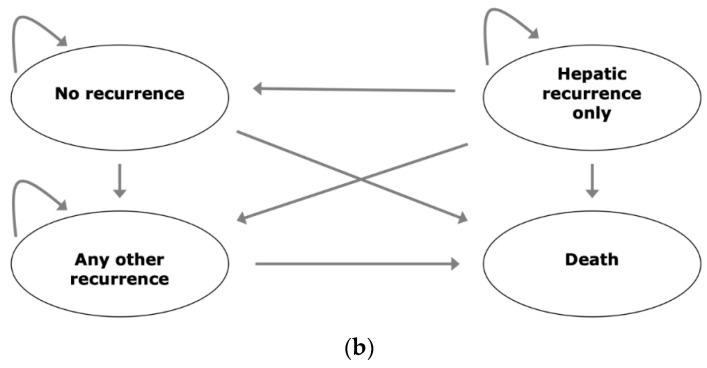
Model overview. (**a**). Decision model for both strategies incorporating CE-CT and 18F-FDG PET/CT. For each outcome, a Markov model analysis was performed; (**b**). Markov model with potential states “No recurrence”, “Hepatic recurrence only”, “Any other recurrence” and “Death”. The first state was determined depending on the outcomes in the decision model.

To evaluate the cost-effectiveness of a follow-up 18F-FDG PET/CT scan after percutaneous ablation of CRLM, a decision model based on a Markov model was developed using a decision-analytic software (TreeAge Pro Version 19.1.1, Williamstown, MA, USA). Markov models can be used to stochastically assess long-term outcomes of patients by estimating the probabilities of diverse states and the likelihood of transition from one state to another. The model is summarized in Figure 5.

### 4.2. Input Parameters

The age at ablation was set to 68 years according to published studies [2]. The discount rate was set to 3% [24]. Willingness-to-pay (WTP) was set to $100,000 per quality adjusted life year (QALY) [24]. A summarized overview can be found on Table 1. The analysis is based on the US healthcare system. Age-specific risk of death was derived from the US Life Tables as the largest available data set.

### 4.3. Efficacy of Treatment Modalities

CE-CT sensitivity and specificity were set to 53.40% and 95.70% based on literature comparing both modalities in one study [12]. 18F-FDG PET/CT sensitivity and specificity were set to 84.60% and 92.40% according to a meta-analysis comparing the applicable imaging modalities [12].

### 4.4. Costs and Utilities

Treatment costs of CE-CT, 18F-FDG PET/CT and percutaneous ablation was collected from Medicare in 2019. In addition to this, the costs for hospital stay and delayed ablation were included in the analysis.

Utilities were collected as quality-adjusted life years (QALYs) in terms of the patients’ health state.

### 4.5. Transition Probabilities

In accordance to the previously described Markov model, probability of metastasis with timely and delayed surgery and without malignancy were considered in the analysis. Further, in terms of the Markov model, probabilities of recurrence, death with recurrence and successful remission were included in the analysis. For the estimation of probability of death without complication, US life tables were also utilized.

### 4.6. Cost-Effectiveness Analysis

According to the WTP and discount rate defined above, the expected QALYs and costs were calculated for a baseline scenario. Furthermore, incremental cost effectiveness ratios (ICER) were estimated.

#### Definitions

Willingness to pay (WTP): WTP is used a threshold parameter in economic statistical models with a focus on health care systems. As a value, the WTP represents the amount the general public of a specific health care system is willing to pay for a certain health benefit considering finite resources in this system.

Incremental cost effectiveness ratio (ICER): The ICER can be used as a marker showing the economic value of a strategy in comparison with an alternate strategy and can be generated with the help of the subsequent formula:

$$ICER=\frac{\left(C_{1}-C_{0}\right)}{\left(E_{1}-E_{0}\right)}$$

$C_{1}$ and $E_{1}$ represent cost and effect of an exemplary strategy 1 and $C_{0}$ and $E_{0}$ cost and effect of an exemplary strategy 0. The ICER then represents the extra costs a strategy causes per QALY in comparison to the second strategy which is being examined.

Sensitivity analysis: Sensitivity analysis is used for the assessment of a model’s overall mathematical uncertainty. Since different values of an input parameter can influence the dependent variable, this analysis can be used to see how distinctive sources of uncertainty can make a contribution to the overall uncertainty of the investigated model. The ICER should therefore only show little variation in these analyses as big variations would suggest a high uncertainty of the carried-out analysis.

Deterministic sensitivity analysis: In deterministic sensitivity analysis a specific (one-way sensitivity analysis) or multiple (multivariate sensitivity analysis) input parameters can be altered within a range.

Probabilistic sensitivity analysis: Probabilistic sensitivity analyses are used in a distribution model with multiple iterations by using variables that are sampled from their respective distributions instead of using a point estimate value.

Cost-effectiveness acceptability curve: Cost-effectiveness acceptability curves are used to display the relationship between an ICER and a cost-effectiveness threshold within a certain range. The curves are influenced by the uncertainty of the model on the respective model.

In accordance to the prevailing literature regarding cost-effectiveness analyses in health care systems, the discount rate was set to 3% in this study and the WTP was set to $100,000 per QALY. Since the expected costs were mainly derived from the US as the biggest and most homogenously available dataset, all costs are displayed in United States Dollar (USD) and the respective QALYs were analyzed accordingly. ICERs were calculated according to the method explained above and as described additional sensitivity analyses were performed to assess the uncertainty and the robustness of the used model. The respective results can be seen in Figure 2 and Figure 3. To generate the described above cost-effectiveness acceptability curves Monte Carlo simulations were done using 30,000 s order samples for various WTP thresholds. The respective results can be seen in Figure 3.

### 4.7. Sensitivity Analysis

To analyze the robustness of the model, deterministic and probabilistic sensitivity analyses were performed [46]. For the latter, a number of 30,000 Monte Carlo iterations were applied. Based on the probabilistic analysis, acceptability curves were estimated.

## 5. Conclusions

In conclusion, our study shows that 18F-FDG PET/CT can be cost-effective for follow-up after percutaneous ablation of CRLM patients.

## Figures and Tables

**Table 1:** Model input parameters.

Name	Estimate	Distribution	Source
Probability of incomplete ablation	30%	β	Minami Y et al., 2013 [9] (8 to 40%)
Probability of recurrence	3.20%	β	Oldenburg et al., 2006 [23]
Expected value at diagnostic procedure	68		Engstrand et al., 2018 [2]
Assumed WTP per QALY	$100,000		Sanders et al., 2016 [24]
Discount rate	3%		Sanders et al., 2016 [24]
Model Period	2 years		Sanders et al., 2016 [24]
Diagnostic test performances			
CE-CT sensitivity	53.40%	β	Samim et al., 2017 [12]
CE-CT specificity	95.70%	β	Samim et al., 2017 [12]
18F-FDG PET/CT sensitivity	84.60%	β	Samim et al., 2017 [12]
18F-FDG PET/CT specificity	92.40%	β	Samim et al., 2017 [12]
Costs (Acute)			
CE-CT	$385.00	γ	Medicare (74,177)
18F-FDG PET/CT	$1375.00	γ	Medicare (78,814)
Ablation costs	$1493.00	γ	Medicare 2018/www.cms.gov
Days in hospital for ablation	4	γ	NG KKC et al., 2017 [25]
Cost of hospital stay (per day)	$2184.00	γ	Henry J. Kaiser Foundation, KFF.org
Total reablation costs	$10,229.00	γ	Medicare
Delayed reablation, additional tests	$13,297.70	γ	Assumption to be 1,3 × as expensive
Costs (Long Term)			
Yearly costs without present cancer	$0	γ	Assumption
Yearly costs with present cancer	$25,000.00	γ	Haug et al., 2014 [26]
Utilities			
QOL after ablation for 1 month	0.95	β	Gazelle et al., 2004 [27]
QOL after >1 month: no recurrence	1	β	Fryback DG et al., 1993 [28]
QOL after >1 month: hepatic recurrence only	0.65	β	Kim et al., 2016 [29]
QOL after >1 month: any other recurrence	0.19	β	Kim et al., 2016 [29]
Death	0		Assumption
Transition probabilities			
Efficacy of reablation	93%	β	Shady W et al., 2018 [30]
Probability of metastases with timely surgery	12%	β	NIH-National Cancer Institute
Probability of metastases with delayed surgery	15%	β	Expert opinion
Probability of recurrence	3.20%	β	Oldenburg et al., 2006 [23]
Probability of death with present recurrence/metastases (per year)	5.44%	β	NIH-National Cancer Institute
Probability of successful complete remission in case of present metastases (per year)	80%	β	Wilkinson et al., 1997 [31]
Risk of death for other reason	(age dependent)	β	US Life Tables 2015 (Arias et al., 2018)
